# Holocene evolution of *Portus Pisanus*, the lost harbour of Pisa

**DOI:** 10.1038/s41598-018-29890-w

**Published:** 2018-08-23

**Authors:** D. Kaniewski, N. Marriner, C. Morhange, M. Vacchi, G. Sarti, V. Rossi, M. Bini, M. Pasquinucci, C. Allinne, T. Otto, F. Luce, E. Van Campo

**Affiliations:** 1 Université Paul Sabatier-Toulouse 3, EcoLab (Laboratoire d’Ecologie Fonctionnelle et Environnement), Bâtiment 4R1, 118 Route de Narbonne, 31062 Toulouse, cedex 9 France; 2 CNRS, EcoLab (Laboratoire d’Ecologie Fonctionnelle et Environnement), 31062 Toulouse, cedex 9 France; 3 Institut Universitaire de France, Secteur Biologie-Médecine-Santé, 103 boulevard Saint Michel, 75005 Paris, France; 4 CNRS, Laboratoire Chrono-Environnement UMR 6249, Université de Franche-Comté, UFR ST, 16 Route de Gray, 25030 Besançon, France; 5 Aix Marseille Université, CNRS, IRD, INRA, Collège de France, CEREGE, Aix-en-Provence, France; 6 University of Exeter, Geography, College of Life and Environmental Sciences, Exeter, EX4 4RJ UK; 7 Università di Pisa, Dipartimento di Scienze della Terra, Via S. Maria 53, 56126 Pisa, Italy; 8 Università di Bologna, Dipartimento di Scienze Biologiche, Geologiche e Ambientali, Via Zamboni 67, 40127 Bologna, Italy; 9 Università di Pisa, Laboratorio di Topografia antica, Dipartimento Civiltà e Forme del sapere, Via Pasquale Paoli, 15, 56126 Pisa, Italy; 10 Normandie Université, Université de Caen, Centre Michel de Boüard, CRAHAM, UMR 6273, Esplanade de la paix, 14032 Caen, France

## Abstract

The ancient harbour of Pisa, *Portus Pisanus*, was one of Italy’s most influential seaports for many centuries. Nonetheless, very little is known about its oldest harbour and the relationships between environmental evolution and the main stages of harbour history. The port complex that ensured Pisa’s position as an economic and maritime power progressively shifted westwards by coastal progradation, before the maritime port of Livorno was built in the late 16^th^ century AD. The lost port is, however, described in the early 5^th^ century AD as being “a large, naturally sheltered embayment” that hosted merchant vessels, suggesting an important maritime structure with significant artificial infrastructure to reach the city. Despite its importance, the geographical location of the harbour complex remains controversial and its environmental evolution is unclear. To fill this knowledge gap and furnish accurate palaeoenvironmental information on *Portus Pisanus*, we used bio- and geosciences. Based on stratigraphic data, the area’s relative sea-level history, and long-term environmental dynamics, we established that at ~200 BC, a naturally protected lagoon developed and hosted *Portus Pisanus* until the 5^th^ century AD. The decline of the protected lagoon started at ~1350 AD and culminated ~1500 AD, after which time the basin was a coastal lake.

## Introduction

While Italy’s rich maritime history has sharpened focus on ancient harbours and human impacts in port basins (e.g. *Altinum*-Venice, *Portus Lunae*-Luni, *Portus*-Rome, *Ostia*-Rome, *Neapolis*-Naples)^1–8^, the evolution of *Portus Pisanus*, the powerful seaport of the city of Pisa (Italy, Tuscany), was, until recently, largely unknown^9–15^. The city is currently located ~10 km east of the Ligurian Sea coast but its long and complex history^16,17^ is closely linked to its port, named *Portus Pisanus* by late Roman sources (Rutilius Namatianus descriptions in *de reditu suo*, *Itinerarium Maritimum 501*)^15,18–20^. Since the Imperial Roman period, most of the supplies imported from Mediterranean provinces reached the city of Pisa through a complex harbour system that included *Portus Pisanus*, the fluvial Ports San Piero a Grado and Isola di Migliarino, and other minor landings along the ancient coast, the Arno and Serchio rivers and canals including the site of Pisa-Stazione Ferroviaria San Rossore^21–26^. The heyday of the seaport played out from the Late Republican Roman period to the Middle Ages, when Pisa became an influential *Commune* and maritime power^16,18,27^. In the Middle Ages, *Portus Pisanus* was the main harbour of Pisa: the harbour system included Vada, the ports of the Piombino promontory, Castiglione della Pescaia and other minor landings^27^. During the 13^th^ century AD, it was one of the most important seaports in Italy, rivaling Genoa, Venice, and Amalfi^16,28–30^.

The name *Portus Pisanus*, documented since the 5^th^–6^th^ centuries AD, was probably in use earlier. In 56 BC, Cicero mentioned a harbour (*Ad Quintum fratrem* 2, 5), *Portus Labro*, probably located in the same area. Is *Portus Labro* an earlier name for *Portus Pisanus* (as might be suggested by the current hydronym Calambrone, situated north of Livorno) or a different harbour? In the Middle Ages, the name *Portus Pisanus* was used to identify both the harbour and a large part of the Livorno hinterland. Between the second half of the 14^th^ century AD and the mid-15^th^ century AD, the names *Portus Pisanus* and Porto di Livorno coexisted. While the appellation Porto di Livorno became prevalent, part of the harbour basin was still named *Portus Pisanus*. The name Porto di Livorno has prevailed since the late 1500 s AD.

The exact location of *Portus Pisanus* has long been discussed, despite several Roman literary sources (e.g. *Itinerarium Maritimum*, *de reditu suo*) and studies^10,31,32^ that placed the harbour ~10–13 km south of the city of Pisa, in an area which presently corresponds to the north-eastern edge of the port city Livorno, and despite an accurate description of the area (Santo Stefano ai Lupi) published by Targioni Tozzetti in 1775^33^ with an associated map. Recent archaeological excavations at Santo Stefano ai Lupi corroborate both the classical sources and Targioni Tozzetti’s description with the discovery of portions of sea bed covered by fragments of ancient pottery (dated to the 6^th^–5^th^ centuries B.C. and to a period between the 1^st^ century BC and the 6^th^ century AD), ballast stones, part of a small stone dock, some buildings including a warehouse, and a necropolis dated to the 4^th^–5^th^ centuries AD^14,34,35^. These structures belong to *Portus Pisanus’* harbour system, but just a small area of the ancient port city and the adjacent basin has been excavated. The pottery fragments found on the ancient sea bed prove that, in this part of what was called *Portus Pisanus*, boats and ships were loaded and unloaded. The associated harbour basin is believed to be a large, naturally sheltered embayment that accommodated ships from the Etruscan and Roman periods to the Late Medieval Ages, when Pisa grew into a very important commercial and naval center, controlling a significant merchant fleet and navy^35^. The existence of a highly protected natural basin would have been of great benefit to navigation and trade, facilitating the establishment of port complexes, through the use of the natural landscape. Historical sources or archaeological excavations have not unearthed any artificial moles at *Portus Pisanus*, suggesting a very confined harbour that was naturally protected by its geomorphological endowments^20^.

Here, we use bio- and geosciences to probe the long maritime history of the seaport of Pisa. We present a 10,500-year Relative Sea-Level (RSL) reconstruction to understand the role of long-term sea-level rise in shaping the harbour basin. We also report an 8000-year reconstruction of environmental dynamics in and around the harbour basin to establish when *Portus Pisanus* became a “naturally sheltered embayment”, reported in literary sources as the main hub of the ancient Pisa harbour system. Finally, we map the location and evolution of the harbour basin since the Roman period. These environmental data were compared and contrasted with the history of Pisa (written sources and archaeological data).

## Results

### RSL reconstruction

**Figure 1:**
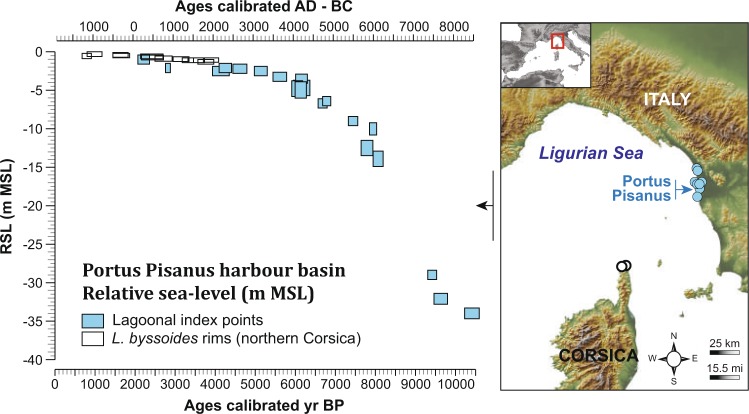
Relative sea-level reconstruction of the eastern Ligurian Sea. The RSL history is based on 31 index points deriving from lagoonal sediment archives of the Arno and Versilia coastal plains and fossil *Lithophyllum byssoides* rims from Northern Corsica. The blue boxes represent index points from lagoons and salt marshes. The black boxes are *L*. *bissoides*-derived index points. The dimensions of the boxes denote the 2 s altitudinal and chronological errors associated with each index point. The map is an original document drawn using Adobe Illustrator CS5 (http://www.adobe.com/fr/products/illustrator.html).

The harbour basin is the outcome of an environmental history in which long-term sea-level rise has played a key role. A total of 31 RSL index points has been used to frame the Holocene sea-level evolution of the eastern Ligurian Sea (Fig. 1). At ~8550 BC, the oldest index points place the RSL at ~35 m below current Mean Sea Level (MSL). Younger index points indicate that RSL rose rapidly until ~5050 BC followed by a slowdown in the rising rates. At ~4050 BC, multiple RSL index points constrain the RSL to ~−5 m MSL. Since this period, index points delineate a significant reduction in the rising rates that become minimal during the last ~4000 years, when the total RSL variation was within 1.5 m MSL. The reconstructed RSL history reflects the pattern observed in the mid to northern portion of the western Mediterranean^36,37^. The significant slowdown in rising rates after ~5550 BC is consistent with the final phase of North American deglaciation^38^ while the further decrease in rising rates is related to the progressive reduction in glacial meltwater inputs that were minimal during the last ~4000 years^39,40^.

### Drilling the harbour basin

**Figure 2:**
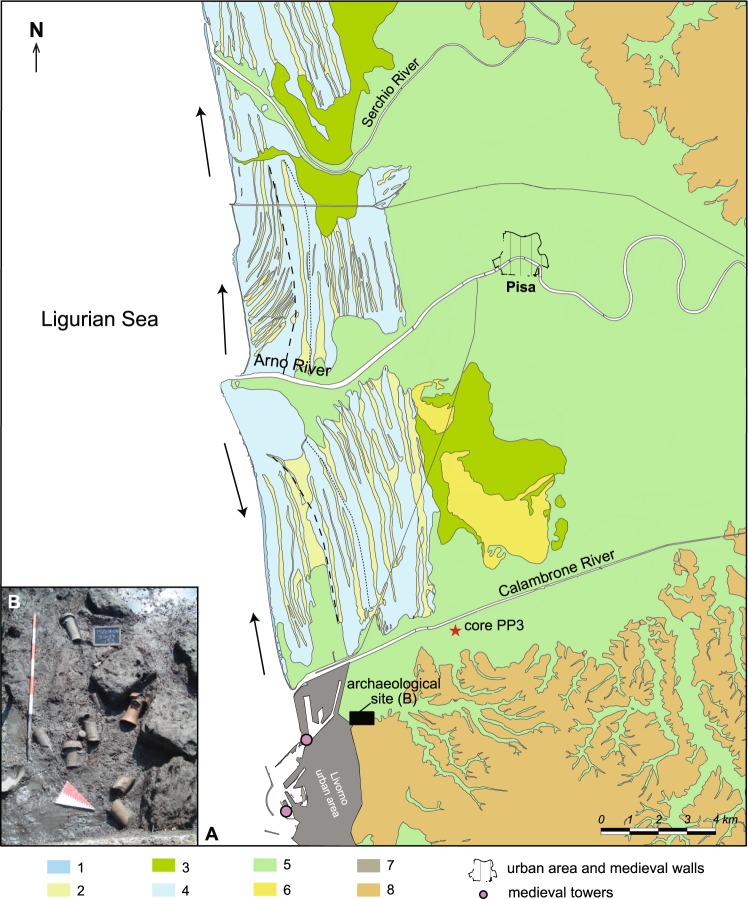
Study area and location of the archaeological site. **(A**) Geomorphological map of the study area (modified from CARG Regione Toscana). **1** current beach; **2** shallow swale; **3** wetland; **4** beach ridge, superimposed dunes; **5** alluvial plain; **6** residual relief; **7** Livorno urban/industrial area; **8** mountains and hills; dashed line: 17^th^ century AD coastline; dotted line: 12^th^ century AD coastline; arrow: current drift. (**B**) Photograph of the archaeological site.

The terrestrial, marine and freshwater biological indicators used to reconstruct palaeoenvironmental evolution of *Portus Pisanus* were extracted from an 890-cm sedimentary core (PP3, 43°35′55.33″N, 10°21′41.71″E; +2 m MSL; Fig. 2) drilled ~5 km from the sea on the southern portion of the modern Arno River alluvial-coastal plain, close to Livorno and the Pisa Hills. During ancient times, this part of the alluvial plain, located ~10 km south of Pisa, was fed by a former branch of the Arno River^41^ here reported as the Calambrone River. According to previous studies^15,18,20^, the sedimentary core was taken from the former harbour area, active from the Archaic Etruscan (6^th^–5^th^ centuries BC) to the Late Roman periods (5^th^ century AD). In the Middle Ages, the port shifted westwards, as testified by the building of the fortified harbour basin of Leghorn including medieval towers dated from 1300 and 1400 AD (Fig. 2), and then again further westwards in modern times, consistent with the progradation dynamics of the delta. The core was recovered behind the innermost beach-ridge of the Arno Delta complex, where a meter-thick back-barrier succession occurs, recording the development and prolonged persistence of a wide lagoon basin during the Late Holocene. Back-barrier deposits, mainly represented by fossiliferous clay-silt interbedded with sandy layers, are overlain by a progradational suite of coastal-alluvial facies, deposited during the recent phase of decelerating sea-level rise.

**Figure 3:**
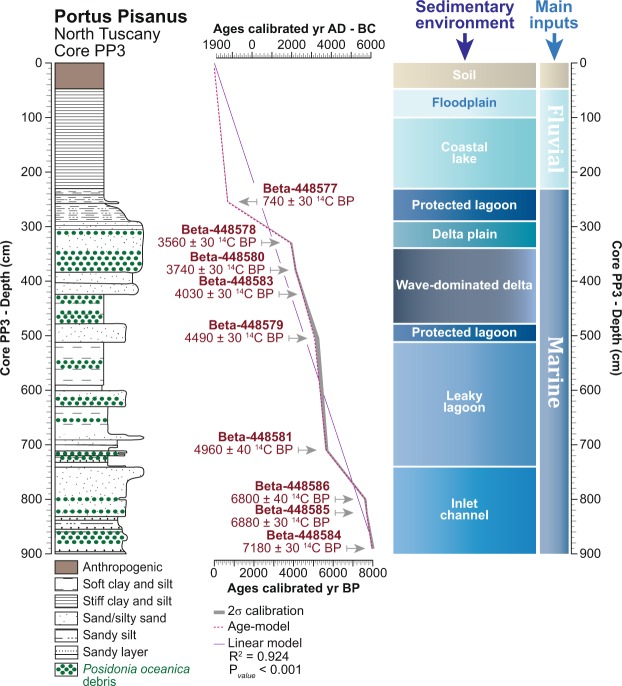
Details of *Portus Pisanus* basin in North Tuscany, Italy. The lithology of the core in the basin area, with the influence of marine components, is reported according to depth. The main sedimentary environments are plotted on a linear depth-scale. The radiocarbon dates are depicted as 2σ calibrations (95% of probability). The age model is superimposed on the 2σ calibration curve, and a linear-model was also added showing a theoretical continuous sedimentation rate. The timescale is shown as BP and BC-AD.

The chronology of the core PP3 is based on nine accelerator mass spectrometry radiocarbon (^14^C) dates (Fig. 3). Dated samples (short-lived terrestrial samples: seeds, small leaves of annual plants) were calibrated [2-sigma (σ) calibrations, 95% of probability] using Calib-Rev 7.1 with IntCal13. According to the ^14^C chronology, the core covers the last 8000 years (Fig. 3). The age model (Fig. 3) was calculated using Xl-Stat^2017^ and Calib-Rev 7.1. The dates obtained in between each ^14^C dating are modeled and therefore are liable to mask some of the temporal variability in the depositional patterns. The calculated model displays an average 2-sigma range of 50 years (P < 0.001) for the whole sequence. All the calibrated ages are shown/discussed as BC/AD to fit with the archaeological-historical data and are presented at the 2-sigma range (95% probability). The two scales, BC-AD and BP, are both displayed on each figure. While the average chronological resolution of the core stratigraphy is 9 years per cm^−1^ (1.11 mm per yr^−1^), a homogeneity test (Monte Carlo simulation, standard test, P_value_ < 0.001) suggests two abrupt changes in the sedimentation rate at 740 cm depth (4300 ± 70 BC) and 290 cm depth (200 ± 30 BC).

### Biological proxies in the harbour basin

**Figure 4:**
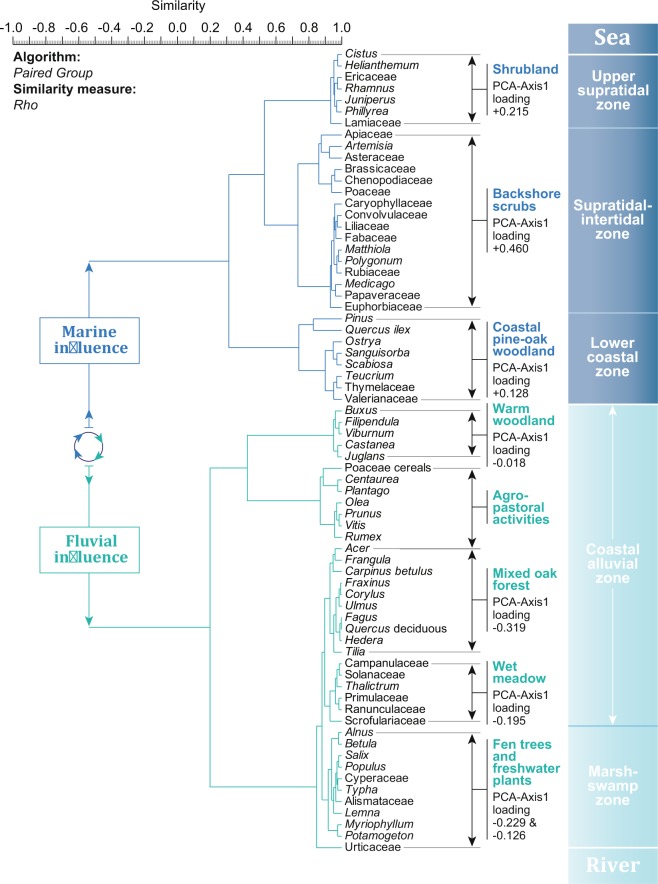
Pollen-based ecological clusters from the harbour basin for the last 8000 years. A cluster analysis (paired group as algorithm, Rho as similarity measure) was used to define the ecological assemblages. Each cluster was summed to create pollen-derived vegetation patterns. The potential location of each cluster, from the intertidal zone to the hinterland, is indicated.

**Figure 5:**
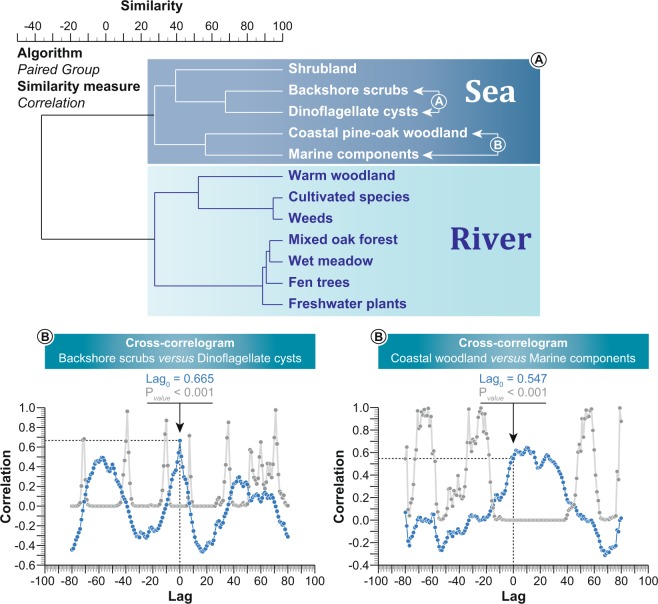
Environmental-based clusters from the harbour basin for the last 8000 years. (**A**) A cluster analysis (paired group as algorithm, Correlation as similarity measure) was used to define the environmental assemblages (marine *versus* fluvial influence). (**B**) The two cross-correlograms (P = 0.05) depict the marine influence on ecosystems around the basin.

Terrestrial data retrieved from *Portus Pisanus’* harbour basin were analysed using a cluster analysis (descending type). Each cluster was summed to generate pollen-derived vegetation patterns and assigned to a potential location, from the intertidal zone to the hinterland (Fig. 4), referring to modern patches of vegetation along the Ligurian coast (local data and Vegetation Prodrome^42^). To ascertain the ordination of terrestrial data according to the “sea” factor, a second cluster analysis (descending type; Fig. 5A) was calculated using the vegetation patterns and the marine proxies [dinoflagellate cysts and marine components (foraminifera, marine bivalves, debris of *Posidonia oceanica*)]. Three vegetation communities (backshore scrubs, shrubland, and coastal pine-oak woodland) are linked to a marine influence (from the supratidal zone to the lower coastal zone; Figs 4–5A) whereas the other communities (mixed oak forest, wet meadow, fen trees, freshwater plants) are related to fluvial inputs from the Calambrone River (coastal alluvial zone and marsh-swamp zone). Cross-correlations (vegetation patterns *versus* marine proxies; Fig. 5B) also indicate that seawater has influenced proximal vegetation patterns (positive correlations on the null lag score: Lag_0_ = 0.665, Lag_0_ = 0.547 and Lag_0_ = 0.307, P = 0.05) around the basin. The vegetation group “warm woodland” is set apart as this cluster is mainly related to a third influence, agro-pastoral activities (Fig. 5A) that are observed in the area after 3350 ± 90 BC. Agriculture is composed of cereals (Poaceae cerealia), olive trees (*Olea europaea*), common grape vines (*Vitis vinifera*), and other trees (*Prunus*). The associated anthropogenic indicators are common weeds (*Centaurea*, *Plantago* and *Rumex*).

### Pre-harbour facies (*sensu* Marriner and Morhange)

**Figure 6:**
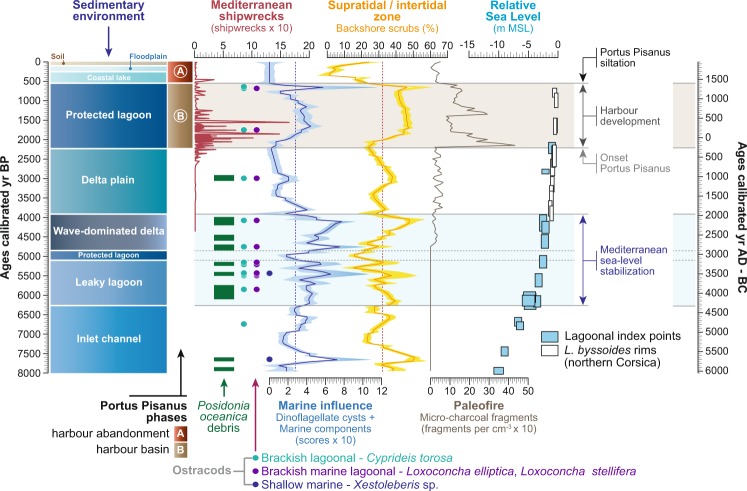
Reconstructed marine influence in *Portus Pisanus* during the last 8000 years. The marine influence (components per cm^−3^) and the backshore scrubs (%) are displayed as a LOESS smoothing (with bootstrap and smoothing 0.05) plotted on a linear timescale (BP and BC-AD). The *Posidonia oceanica* debris (presence/absence) are indicated by green marks along the marine curve. The ostracods (presence/absence) are displayed as dots. Fire activity in the area is shown as charcoal concentrations (fragments per cm^−3^) plotted on a linear timescale. The relative sea level, displayed as MSL, is depicted for the last 8000 years. The shipwreck curve from the Mediterranean region is also plotted on a linear timescale^76^. The brown-shaded horizontal section indicates the harbour development and the blue-shaded horizontal section shows the period when the sea-level stabilized.

**Figure 7:**
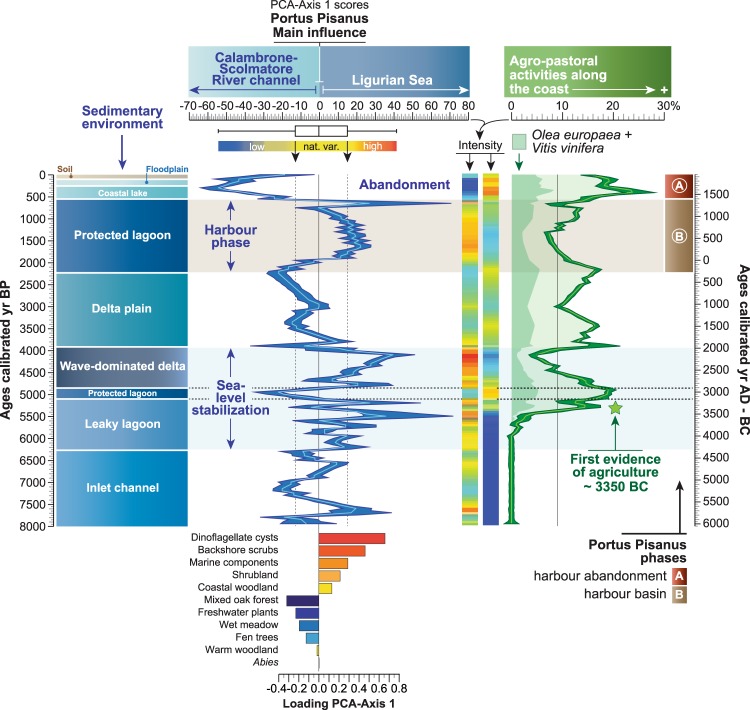
Reconstructed environmental dynamics in *Portus Pisanus* during the last 8000 years. The PCA-Axis1, plotted on a linear age scale (BP and BC-AD), is displayed as a LOESS smoothing (with bootstrap and smoothing 0.05) and a matrix plot. A boxplot was added to mark the extreme scores. The loading of each cluster is indicated below the PCA-Axis1 curve. The agro-pastoral activities (agriculture and weeds, %) are plotted on a linear age scale, and also displayed as a matrix plot. The brown-shaded horizontal section indicates the harbour phase and the blue-shaded horizontal section shows the period when sea-level stabilized.

Marine *versus* fluvial influence since 8000 years is represented by the importance of marine indicators (dinoflagellate cysts and marine components) and supratidal-intertidal scrubs in and around the pre-harbour (Fig. 6). A principal components analysis (PCA) was run to test the ordination of ecosystems by assessing major changes in the area including vegetation patterns, dinoflagellate cysts and marine components (Fig. 7). Environmental dynamics (marine *versus* fluvial influence) in the basin is indicated by the axis-1 of a principal components analysis (PCA-Axis1). The PCA-Axis1 (61% of total variance) is positively loaded by vegetation patterns indicative of a saline-xeric environment, dinoflagellate cysts and marine components. The negative scores correspond to freshwater vegetation types (Fig. 7). The PCA-Axis1 reflects the ecological erosion of wetlands by the intrusion of seawater into the freshwater-fed plains, raising salinity in the hinterland, with land fragmentation and salt-water intrusion into the groundwater table, in and around the basin. The PCA-Axis1 can be considered as a proxy for marine ingression in/around the pre-harbour (with a main physical impact and several secondary influences such as salt spray and salinization)^43^.

**Figure 8:**
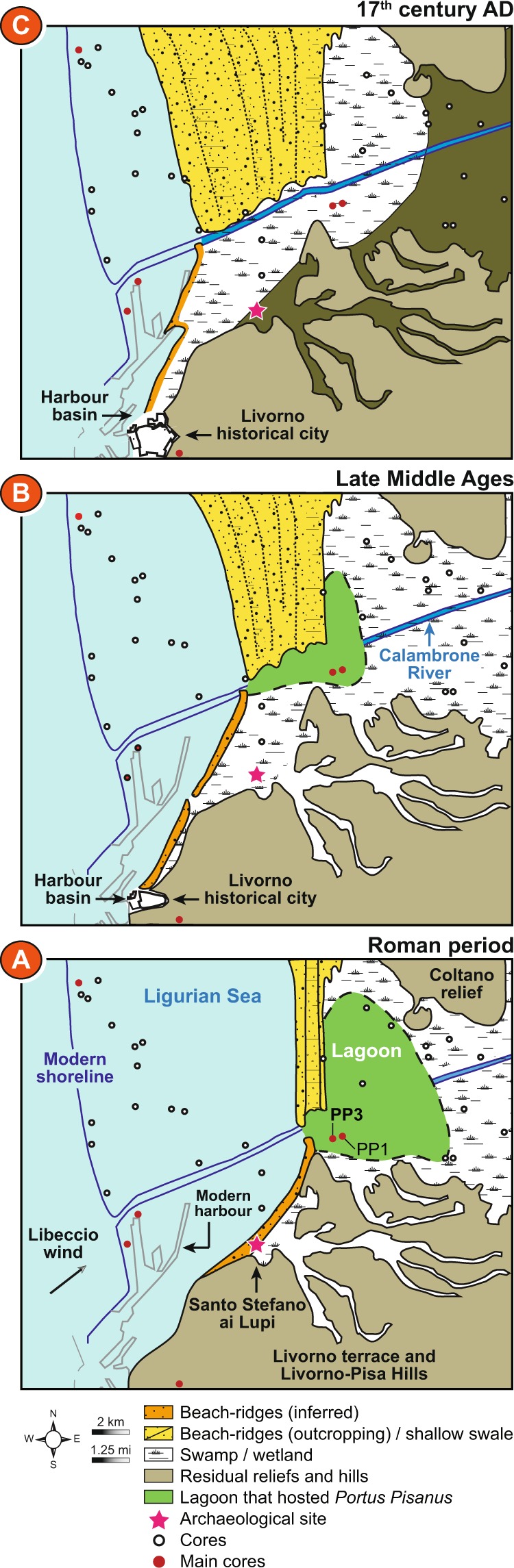
Geographic maps showing three evolutionary stages of the lagoon, in relation to *Portus Pisanus* as documented by historical sources and archaeological data. Maps were produced by integrating stratigraphic (cores and trenches) and geomorphological data (Pranzini, 2007) with historical cartography. Dots indicate cores used to draw the maps (key cores are highlighted by red dots). The modern shoreline is depicted on each map for reference. The grey arrow indicates the direction of the predominant wind (*Libeccio*). (**A**) Roman period - a wide lagoon basin, hosting *Portus Pisanus* as mentioned in literary sources; (**B**) late Middle Ages - the accretion of arcuate beach ridges, belonging to the Arno Delta strandplain, led to an increase in the degree of confinement of the lagoon basin. Construction of the maritime harbour of Livorno in a seaward position with respect to the lagoon; (**C**) 17^th^ century AD - the rapid accretion of strongly arcuate sets of beach ridges led to the siltation of the lagoon that was transformed into a wetland, physically detached from the Ligurian Sea. *Portus Pisanus* abandonment and expansion of the fortified maritime harbour of Livorno.

While permanent inputs of seawater and fluvial freshwater were recorded in the pre-harbour, occurrences of higher marine influence are locally underlined by a combination of peaks in marine indicators, the occurrence of different ostracod ecological groups (shallow marine; brackish-marine and euryhaline), important increases in *Posidonia oceanica* debris (Fig. 6), and strong positive deviations in the PCA-Axis1 scores (Fig. 7). Our reconstruction shows two early periods of seawater influence (at 5800 ± 40–5425 ± 55 BC and 4750 ± 60–4500 ± 60 BC) when the site was a marine invagination, followed by an unstable phase when sea-level stabilized along the Tuscany coastline (Fig. 1), between 4250 ± 60 BC and 2000 ± 45 BC (Fig. 8). Potential discontinuities in environmental dynamics were assessed using a homogeneity test on the PCA-Axis1 (Monte Carlo simulation, Pettitt and Buishand tests). The outcome indicates that the environmental dynamics are not uniform, underlining a major break around 2000 ± 45 BC (P_value_ < 0.001). A second homogeneity test, only applied to the period 3350–6050 BC, highlights a second important break around 4250 ± 60 BC (P_value_ < 0.001, Monte Carlo simulation, Pettitt and Buishand tests), when the pre-harbour evolved into a leaky lagoon. During this period (4250 ± 60–2000 ± 45 BC), the last main peak in the PCA-Axis1 corresponds to a wave-dominated delta and occurred within the chronological interval of the 4.2 ka BP event^44^, suggesting the potential role of climate in influencing the pre-harbour’s evolution. A later phase was recorded at 1250 ± 40–850 ± 40 BC when the pre-harbour evolved into a delta plain, corresponding to the 3.2 ka BP event^45,46^.

Among the main periods of marine influence in the harbour basin, before the establishment of *Portus Pisanus*, the period 4250 ± 60–2050 ± 45 BC was the hinge phase, culminating in a wave-dominated delta. These periods, characterized by inland salt intrusion, significantly affected the agricultural productivity of this coastal area (Fig. 7). Prolonged marine inundation appears to have led to the salinization of agriculturally productive soils resulting in diminished output for long periods of time (Fig. 7).

### *Portus Pisanus*

Because the area has been frequented by archaic ships since at least the 6^th^–5^th^ centuries BC, the first phases of navigation had to be carried out on a delta plain characterized by wetlands^34,35^. According to our reconstruction (Figs 6–7), marine influence increased in the harbour basin after 200 ± 30 BC, when a naturally protected lagoon developed and hosted *Portus Pisanus* up to the 5^th^ century AD (according to archaeological evidence^14,34,35^). During this period, a first peak in charcoal fragments is recorded at 180 ± 30 BC. A second inflection in charcoal fragments occurred at 550 ± 25 AD and is synchronous with a major fall in agro-pastoral activities (Fig. 7). From 1000 ± 20 to 1200 ± 20 AD, a first decrease in marine influence was documented before the last major marine phase at 1300 ± 20 AD. The decline of the protected lagoon started at 1350 ± 15 AD and ended at 1500 ± 10 AD, when the basin evolved into a coastal lake, concomitant with the development of agriculture, then to a floodplain (1700 ± 10 AD) and finally a soil atop a fluvial plain (1850 ± 5 AD).

**Figure 9:**
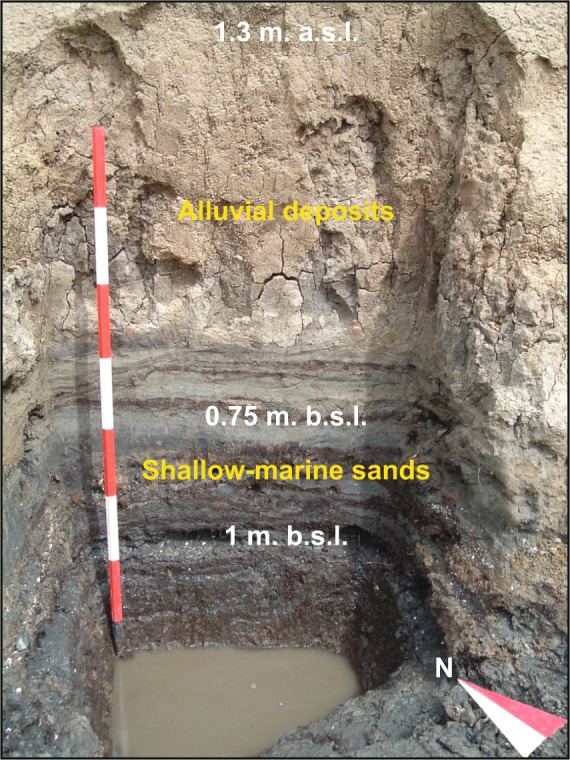
Stratigraphic trench from the archaeological site of Santo Stefano ai Lupi. Representative photograph depicting the stratigraphy of the archaeological trench excavated at the Santo Stefano ai Lupi site (see Fig. 2 for location). The lower portion of the section consists of shallow-marine sands rich in *Posidonia* fibers, mollusc shells/fragments and ceramics of Roman age. These sands grade abruptly upwards into fine-grained alluvial deposits. Depths are displayed leveling relation to MSL.

Stratigraphic data from several cores (Fig. 8) and archaeological trenches (Fig. 9) undertaken at Santo Stefano ai Lupi (Fig. 2), along with prominent geomorphological features (e.g. outcropping beach ridges and residual reliefs), were used to produce three maps, which illustrate the landscape evolution of the southern portion of the Arno alluvial-coastal plain, between the Roman period and the Modern age (Fig. 8). The extension and the environmental characteristics of *Portus Pisanus* are based on facies correlations (chronological framework based on ^14^C dates and archaeological data). The core PP3 (and also PP1) formed the type stratigraphy of the basin (Fig. 8).

## Discussion

Recent archaeological and palaeoenvironmental studies at Pisa have focused both on *Portus Pisanus*^14,15^ and the ancient fluvial port in the area of Pisa-Stazione Ferroviaria San Rossore, where several ships dating from the 2^nd^ century BC to the 5^th^ century AD were discovered^21–25^. These exceptional findings (amphorae, artefacts pertaining to the loads carried, and on-board equipment such as sails, ropes, anchors) engendered a wider environmental study focused on the analysis of Arno flood events in relation to the shipwrecks^26,47^ and on archaeobotany^5^.

Although the study of the Pisa-Stazione Ferroviaria San Rossore fluvial port is imperative for improving our knowledge of both the Pisa territory and its commercial activities, *Portus Pisanus* was the city’s main harbour, centered on Mediterranean-wide trade. This port witnessed the rise and fall of Pisa. The lagoon, which hosted the seaport, has recorded the long environmental history of the coast before turning into a protected lagoon and becoming one of the most important natural harbours in the western Mediterranean.

### Before the environment becomes a harbour basin

*Portus Pisanus* and the related lagoon are the result of a long and complex environmental history (Figs 6–7), leading to the development of a lagoon characterized by a narrow entrance (Fig. 8). The base of this ancient lagoon corresponds to a long and narrow marine invagination (inlet channel) that developed to the south of the city of Pisa from 6000 ± 35 to 4300 ± 70 BC. The formation of the channel resulted from sea-level rise from −10 to −4.5 meters below MSL (Fig. 1), with two phases of stronger marine influence at 5800 ± 40–5425 ± 55 BC and 4750 ± 60–4500 ± 60 BC (Fig. 6). No evidence of agro-pastoral activities are recorded during this first stage, suggesting the absence of human impacts on the coastal area (Fig. 7).

Around 4250 ± 60 BC, the inlet channel evolved into a leaky lagoon (Figs 6–7) as the sea-level rose from −4.5 to −2 meters below MSL (Fig. 1). As a consequence, the area was characterized by recurrent seawater inputs of increasing intensity (Fig. 6). The emergence of agriculture is dated to 3350 ± 90 BC (Fig. 7), consistent with nearby coastal sites such as Lago di Massaciuccoli (northwest Tuscany^48,49^) and Stagno di Maccarese near Rome^50^.

The hinge phase of marine influence recorded by a prolonged wave-dominated delta period is centered on 2150 ± 45 BC and may fit with the 4.2 ka BP event^44,51,52^. This climate event corresponds to a stronger seawater imprint in the basin area probably due to decreasing precipitation^53,54^ and weaker fluvial inputs^55,56^. As a consequence, agro-pastoral activities also declined around the basin, with the lowest scores centered on 2150 ± 45 BC (Fig. 7). The event ended at 1950 ± 45 BC (Fig. 7) and the basin was transformed into a delta plain characterized by higher inputs of freshwater (Fig. 6).

The last phase of marine influence before the emergence of the lagoon that hosted *Portus Pisanus* occurred at 1250 ± 40–850 ± 40 BC, but with a weaker intensity compared to the 4.2 ka BP event (Fig. 7). This deviation may correspond to the 3.2 ka BP event^45,46,57^, a dry spell that favored marine inputs into the basin as a result of decreasing river flow (Fig. 7). In Italy, this event is characterized by reduced precipitation (Buca della Renella cave, central-western Italy^53^) and a drop in lake levels^58^. At the end of this event, river flow increased until 200 ± 30 BC, when a large, naturally sheltered lagoon with a good connection to the sea, developed and hosted *Portus Pisanus*.

### The lagoon as a mirror of *Portus Pisanus*

Although the area has been settled since the late Iron Age, Pisa only became a city in the archaic period (7^th^–6^th^ centuries BC). According to archaeological evidence, the earliest navigation and port activities recorded in what later became *Portus Pisanus* are documented by Greek, Etruscan and local pottery fragments scattered on the lagoon floor and dated to the end of the 6^th^ or early 5^th^ century BC (Fig. 6). The first evidence of Roman harbour infrastructure in the *Portus Pisanus* area date back to the 2^nd^ century BC^34,35^ when the rate of shipwrecks intensifies in the Mediterranean, suggesting a sharp increase in maritime trade (Fig. 6). The environmental reconstruction indicates that the onset of the protected lagoon (Fig. 8) is chronologically constrained to around 200 ± 30 BC (Figs 6–7). A break in sedimentary deposition (Fig. 3), as well as the development of marine-influenced ecosystems (Fig. 7) and the emergence of typical lagoonal ostracod fauna^20^, dominated by *Cyprideis torosa* (Fig. 6), attest to a changing environment, characterized by a shift towards a calmer basin (Fig. 3). This evolution suggests that the harbour basin constitutes a natural lagoon with a good connection to the sea, consistent with historical sources that mention, in the early 5^th^ century AD, “a large, naturally sheltered embayment” (*de reditu suo*, *1*, *527-540*; *2*, *11*-*12*). A first peak in fire activity at 180 ± 30 BC (Fig. 6) may correspond to the period when the Ligures repeatedly set fire to Pisan crops and countryside^10^. The important development in agriculture (Fig. 7), marks the period when Pisa was *romanised*^17^. While the growth in population during the Roman period, as well as the development of trade and shipbuilding, favored by *Portus Pisanus*, the *Via Aurelia* and the *Via Aemilia Scauri*, resulted in an extension of the inhabited area^17^, agro-pastoral activities around the basin decline (Fig. 7). The first decline of Pisa is documented during the 5^th^ and 6^th^ centuries AD, especially during the Gothic War (~550 AD). A second peak in fire activity and a strong decline in agro-pastoral activities at 550 ± 25–600 ± 25 CE probably resulted from destruction wrought by the Gothic War. Nonetheless, the seaport continued to be active after the fall of the Roman Empire (476 AD). For instance, Pisa remained an important harbour city for Goths, Longobards and Carolingians (from 493 to 812 AD)^28,59^. No major environmental changes were recorded in the harbour basin during this period, suggesting the presence of a natural deltaic coastal spit protecting the environment (Fig. 7). The first phase of decreasing seawater inputs (1000 ± 20–1250 ± 20 AD), due to increasing coastal progradation processes, corresponds to the period when Pisa became a powerful *Commune* and when the fortified medieval harbour of Livorno was built to the west of the Roman port^60,61^. Part of what once was the Roman *Portus Pisanus* remained a protected lagoon basin but with a lower degree of sea connection (Figs 6–7). Agro-pastoral activities increased after 900 ± 25 AD (Fig. 7), probably favored by the extension of wetlands (Fig. 8).

In the late 13^th^ century AD, when the last peak of marine influence was recorded (1300 ± 20 AD; Fig. 7), the defeat of Pisa’s navy in the Battle of Meloria against Genoa (1284 AD) marked the onset of the city’s declining power. In 1290 AD, Genoese ships attacked the Medieval *Portus Pisanus*, sealing the fate of the independent Pisan state. Livorno was finally sold to Florence in 1421 AD^60,62^ when the former Roman harbour basin started to lose its direct connection with the sea (Fig. 6) due to silting and coastal progradation (Fig. 8). The juxtaposition of strongly arcuate sets of beach ridges is consistent with a pronounced increase in fluvial inputs^63^. At 1500 ± 10 AD, the lagoon was cut off from the sea and replaced by the maritime harbour of Livorno located on a rocky coast beyond the deltaic system (Fig. 8). The decision to build two new docks in this port was taken by Cosimo I Medici in 1573 AD. The old basin was then transformed into a coastal lake (from 1400 ± 15 to 1700 ± 10 AD; Figs 6–7) and then into a floodplain (from 1700 ± 10 to 1850 ± 5 AD). During these latter periods, the highest peaks in agriculture were recorded at 1550 ± 10–1600 ± 10 AD (Fig. 7), when Pisa was under the influence of the Duchy of Florence and the Grand Duchy of Tuscany.

### Catastrophic flood events

A comparison with “catastrophic flood events” recorded at Pisa^47^ shows that the hinge phases^63,64^ correspond to periods of minor decreases in marine influence in the harbour basin, suggesting a weak impact of these hydrological events on the seaport. A weak agro-pastoral signal during the period 50 ± 30 BC-900 ± 25 AD (Fig. 7) is probably not the outcome of recurring floods, which would have affected human activities, but is likely the result of small cultivated fields in a marshy deltaic environment.

## Conclusions

Bio- and geosciences have unraveled the complex history of the protected lagoon that hosted *Portus Pisanus*, shaped by relative sea-level changes, coastline variations, fluctuations in river discharge and sediment supply, climate and human impacts. The site where the harbour complex was located was both its strength and its weakness because, like other deltaic contexts^65,66^, sediment supply eventually entrained its demise. *Portus Pisanus* was destined to disappear due to long-term coastal dynamics and environmental change.

## Methods

### Relative sea-level reconstruction

We built a database of RSL index points (i.e. a point that constrains the palaeo mean sea-level in space and time^67,68^) for the eastern Ligurian Sea (Fig. 1). We followed the recent protocol proposed by Vacchi *et al*.^36^ for the Mediterranean Sea. Index points were mainly produced from radiocarbon-dated samples of brackish lagoonal sediments collected near *Portus Pisanus* (i.e. the Arno and Versilia coastal plains, Fig. 1). We further added a suite of radiocarbon-dated samples deriving from fossil *Lithophyllum byssoides* rims in Northern Corsica^69^. All the radiocarbon ages were calibrated into sidereal years with a 2σ range using Calib-Rev 7.1. We employed the IntCal13 and Marine13 datasets for terrestrial and marine samples, respectively. The indicative range (i.e. the relationship of the samples with respect to the former mean sea level) of each RSL index point was established according to Vacchi *et al*.^36^.

### Maps

Different surface and subsurface datasets were used to reconstruct the late Holocene palaeoenvironmental evolution of the *Portus Pisanus* area^15,19^. A geomorphological survey complemented by remote sensing analysis (satellite images, multitemporal photographs, LiDAR images) was undertaken to identify outcropping beach ridges and to verify data previously proposed by other authors^70,71^. In order to accurately reconstruct changes in coastal morphology and place constraints on the location and size of Leghorn port structures during the Modern Age (between the 16^th^–17^th^ centuries AD), historical maps^72,73^ were georeferenced and coupled with the geological data.

In a GIS environment, the geomorphological features were matched with stratigraphic subsurface reconstructions based on facies correlations and geometric criteria. Subsurface data include archaeological trenches from the Santo Stefano ai Lupi site^35^, the highest quality stratigraphic descriptions available for the Arno coastal plain^74,75^ and two reference cores (9 m-long PP1 and PP3 cores) obtained using percussion drilling equipment (Atlas Copco, Cobra model, equipped with Eijkelkamp samplers). The latter were analysed for their sedimentological features (e.g., mean grain size, colour, plant debris, wood fragments) and the fossil content (i.e., benthic foraminifera and ostracods, palynomorphs).

The chronological framework is based on 9 radiocarbon dates performed at Beta Analytic Inc. (Miami, USA) and CIRCE Laboratory of Caserta (Naples University). Further control points were provided by archaeological material from the Santo Stefano ai Lupi site and on the surface of the Arno delta plain^35^.

### Biological indicators

Sampling was done according to the different depositional layers. On average, this corresponds to one sample every 10 cm, but with some variations to respect the core stratigraphy (Fig. 3). All the samples from the core PP3 were prepared for pollen analysis using the standard procedure for clay samples. Pollen grains were counted under x400 and x1000 magnification using an Olympus microscope. Pollen frequencies (expressed as percentages) are based on the terrestrial pollen sum, excluding local hygrophytes and spores of non-vascular cryptogams. Aquatic taxa frequencies were calculated by adding the local hygrophytes-hydrophytes to the terrestrial pollen sum. Dinoflagellate cysts (marine plankton) were counted on pollen slides and are reported as concentrations (cysts per cm^−3^). The fire history was elucidated by counting the pollen-slide charcoal particles (50–200 mm) and is expressed as concentrations (fragments per cm^−3^). Concentrations have been plotted on a linear depth-scale. Foraminifera, marine bivalves and *Posidonia oceanica* debris were extracted from the same samples as the pollen grains, charcoal fragments and dinoflagellate cysts in order to avoid any analytical bias. These marine components (Foraminifera, marine bivalves) and *P*. *oceanica* debris were picked from the washed sediment fraction. The marine components are displayed as concentrations (scores: remains per cm^−3^; Fig. 6). Ostracods were extracted from the core PP1 (Fig. 8) and correlated to PP3 using depth and stratigraphy.

The potential effect of different depositional layers (silty clay *versus* sand) on the conservation of bioindicators (mainly pollen) was also tested using the pollen sums, n-scores, and Simpson index. The data do not show a direct correlation between variations in depositional layers (Fig. 3) and the conservation of bioindicators, suggesting that taphonomic processes, which could affect the signal, are not significant. The whole data set is available in the “Raw data file”.

### Statistical analyses

All data were analysed using Xl-Stat^2017^ and the software package PAST, version 2.17 c. A regular interpolation (50-yr) was first run on the dataset. Biological data and charcoal fragments were analysed using cluster analysis (descending type; Figs 4–5A). Cross-correlations (P = 0.05) were subsequently calculated (Fig. 5B). Cross-correlations concern the time alignment of two time series by means of the correlation coefficient. The time series were cross-correlated to ascertain the best temporal match and the potential delay between the two time-series. The outcome is plotted as a function of the alignment position, focusing on the Lag_0_ value. A LOESS smoothing (with bootstrap and smooth 0.05) was applied to the “marine influence” and backshore scrubs to define the 2.5 percentile and the 97.5 percentile (Fig. 6). Use of the LOESS curve is better than the percentage/concentration-curve to show long-term trends because it is a non-parametric regression method that combines multiple regression models in a k-nearest-neighbor-based meta-model. The LOESS smoothing is here plotted on a linear timescale (Fig. 6). A principal components analysis (PCA) was then run to test the ordination of ecosystems by assessing major changes in the matrix including pollen-derived vegetation patterns, dinoflagellate cysts and marine components (Fig. 7). The “agro-pastoral activities” assemblage (Fig. 4) was excluded from the matrix (Fig. 7). The main variance is loaded by the PCA-Axis1, which is also shown as a LOESS smoothing (with bootstrap and smooth 0.05) plotted on a linear timescale. A boxplot was added to separate the natural variability from the extreme values. Matrix plots are also displayed to mark the hinge phases (Fig. 7).

### Data availability statement

All data generated during this study are included in this article (“Raw data file”) or are available from the corresponding author upon request.

## Electronic supplementary material

Raw data
